# A literature review on surgery for cervical vagal schwannomas

**DOI:** 10.1186/s12957-015-0541-6

**Published:** 2015-03-29

**Authors:** Giuseppe Cavallaro, Giada Pattaro, Olga Iorio, Marcello Avallone, Gianfranco Silecchia

**Affiliations:** Department of Medico-Surgical Sciences and Biotechnologies, Sapienza University of Rome, Corso della Repubblica 79, 04100 Latina, LT Italy

**Keywords:** Schwannoma, Vagal nerve tumor, Cervical schwannoma, Vagus

## Abstract

Cervical vagal schwannoma is a benign, slow-growing mass, often asymptomatic, with a very low lifetime risk of malignant transformation in general population, but diagnosis is still a challenge. Surgical resection is the treatment of choice even if its close relationship with nerve fibres, from which it arises, threats vagal nerve preservation. We present a case report and a systematic review of literature. All studies on surgical resection of cervical vagal schwannoma have been reviewed. Papers matching the inclusion criteria (topic on surgical removal of cervical vagal schwannoma, English language, full text available) were selected. Fifty-three patients with vagal neck schwannoma submitted to surgery were identified among 22 studies selected. Female/male ratio was 1.5 and median age 44 years. Median diameter was 5 cm (range 2 to 10). Most schwannoma were asymptomatic (68.2%) and received an intracapsular excision (64.9%). Postoperative symptoms were reported in 22.6% of patients. Cervical vagal schwannoma is a benign pathology requiring surgical excision, but frequently postoperative complications can affect patients lifelong, so, surgical indications should be based carefully on the balance between risks and benefits.

## Review

The term ‘schwannoma’, first introduced in 1935 by Stout, identifies a benign tumour with sporadic malignant degeneration arising from cranial, peripheral and autonomic nerve sheath cells [1]. It represents 5% of all benign soft tissue tumours [1], equally affects both genders and typically shows higher incidence between the third and fifth decade [2,3]. Head and neck localization is infrequent, and vagal origin in that region is unusual [1,3,4].

Diagnosis is still challenging cause of the numerous pathologies the schwannoma can be confused with (see the displayed list below) [3,5].

Differential diagnosis from schwannoma:

- Paraganglioma
- Branchial cleft cyst
- Inflammatory adenopathies
- Malignant lymphoma
- Metastatic cervical lymphoadenopathies
- Submandibular salivary gland tumours
- Carotid artery aneurysm

Cervical schwannoma typically presents as a slow-growing asymptomatic mass, well circumscribed and encapsulated [3], having an enlargement of 2.5 to 3 mm per year [6], always intimately adherent to its nerve of origin [7] and displacing the internal jugular vein laterally and the carotid artery medially.

Hoarseness is the most common specific symptom due to vocal cord palsy, whereas the pathognomonic sign for vagal schwannoma is a paroxysmal cough during palpation of the mass [3] due to vagal stimulation.

Malignancy is rarely reported and mainly associated to neurofibromatosis type 1 [7-9].

Surgical resection remains the treatment of choice since the very first experiences [10-13], even vagal nerve injury is still an unsolved problem, given the fact that the tumour originates directly from the nerve fibres.

The authors systematically reviewed the literature from the first reports in 1948 in order to identify all the studies in which surgical removal of neck vagal schwannomas was described. Keywords for the research were cervical/neck schwannoma/neurinoma/neurilemmoma, vagus/vagal nerve. All articles were reviewed and those which matched the inclusion criteria were selected. Inclusion criteria were surgical treatment of cervical vagus nerve schwannomas, English language and full text available; papers on nonsurgical treatment of cervical vagus nerve schwannomas, with a language different from English, or with nonavailable full text were excluded.

In each study, demographic and clinical characteristics of patients, schwannomas and related symptoms description, surgical procedures and postoperative outcomes were evaluated when reported and compared.

Among a total of 175 articles listed by PubMed, 153 were excluded because they did not fit the inclusion criteria. Finally, 22 studies were recruited for this review. Fifty-three patients with vagal neck schwannoma submitted to surgery were therefore identified. Women were more frequently affected with a female/male ratio of 1.5 on 35 patients with available data on gender. The onset was estimated on the third and fourth decade in 50% with a median age at diagnosis of 44 years (range 10 to 80). All demographic characteristics are reported in Table 1. Among reported location, left-sided lesions showed a higher frequency than right-sided ones (57.7% *vs*. 42.3%). Side was unavailable in 27 patients. Maximum diameter ranged between 2 and 10 cm with a median value of 5 cm. Most schwannomas were asymptomatic (68.2%); hoarseness was present in four patients (18,2%). Syncope was described just in one patient (4.6%). All patients included in these studies were surgically treated.

**Table 1:** **Patients’ demographic characteristics**

**Demographic characteristics**	**Values**	
Female/male ratio	21/14	1.5
Median age (years)	44	Range (10 to 80)
Side *n* (%)	Right	15 (57.7)
	Left	11 (42.3)

Intracapsular enucleation was the technique of choice by the majority of surgeons (64.9%); about one third were extracapsular resections (32.4%) whereas debulking of tumour was described in just one patient (2.7%). No description of surgical technique was present in 24 cases. Almost the entire cohort of patients was approached through a transcervical incision (93.2%) apart from two who received a postauricular or a transaxillary approach for aesthetic reasons (3.4% each). Six studies (with a total of 24 patients) did not specify the site of incision.

Vagus nerve has been resected in four patients, but just in one, it has been reported a reconstruction with an end-to-end anastomosis. In three cases, a malignant transformation was observed: two angiosarcomas arising within the schwannoma (one of which in a patient with neurofibromatosis type 1) and one case of neurogenic sarcoma. About 25% of patients did not report postoperative complications (26.4%); the predominant postoperative symptom was hoarseness (22.6%), associated or not to other less frequent ones (coughing, chocking when eating, secretions, facial palsy, Horner’s Syndrome).

In about half of patients, postoperative symptoms were not described. Vocal cord palsy was directly confirmed with a laryngoscopy in 19 patients (35.8%), 9 of whom (47.4%) had a complete or partial recovery and 7 (36.8%) a permanent injury. In three cases, no information was available about follow-up. Dividing patients by surgical procedure into two groups (intracapsular excision *vs*. extracapsular resection), it emerged that postoperative complications, such as nerve injury and consequent vocal cord palsy, were present at the same percentage of patients (41.7%), as well as their recovery (60%) suggesting that the choice of surgical technique does not affect outcomes. The small number of patients, however, does not support statistical evidence. Median follow-up of all the series was 9 months (range 3 to 60). No recurrence was observed. Surgical features, procedures, and outcomes are summarized in Table 2.

**Table 2:** **Surgical features**

**Author**	**Number of patients**	**Surgical procedure**	**Approach**	**Preoperative symptoms**	**Vagus resection**	**Postoperative symptoms**	**Complete recovery**
Hwang [15]	1	Extracapsular resection	Transaxillary	−	−	+	+
Ogawa [16]	1	Extracapsular resection	Transcervical	−	+	+	−
Yasumatsu [17]	10	5 extracapsular resections	Transcervical	n.r.	−	n.r.	n.r.
		5 intracapsular excisions					
Gibber [6]	1	Intracapsular excision	Transcervical	−	−	−	
Bilancia [18]	1	Intracapsular excision	Transcervical	+	−	+	−
Chai [19]	1	Intracapsular excision	Transcervical	+	n.r.	+	−
Lahoti [3]	4	n.r.	n.r.	n.r.	−	2 + 2−	n.r.
Lee [9]	1	Extracapsular resection	Transcervical	−	−	+	−
Liu [7]	6	n.r.	n.r.	n.r.	n.r.	n.r.	n.r.
Sreevatsa [20]	3	Intracapsular excision	n.r.	−	−	−	−
Kim [21]	6	Intracapsular excision	n.r.	−	−	1 + 5−	n.r.
Chiofalo [22]	1	Extracapsular resection	Transcervical	+	+	+	−
Bahayani [24]	1	Debulking	Transcervical	−	+	+	−
Peyvandi [4]	1	Extracapsular resection	Transcervical	−	−	n.r.	n.r.
Biswas [1]	2	n.r.	n.r.	1 + 1 n.r.	−	+	+
Kang [25]	6	Intracapsular excision	Transcervical	n.r.	−	3 + 3−	+
Lagner [2]	3	n.r.	n.r.	n.r.	n.r.	n.r.	n.r.
Moroni [26]	1	Extracapsular resection	Transcervical	+	−	−	n.r.
Roh [27]	1	Intracapsular excision	Postauricular	−	−	−	
Matsuo [23]	1	n.r.	Transcervical	+	−	+	−
Ruckert [5]	1	Extracapsular resection	Transcervical	n.r.	+	n.r.	−
Personal Experience	2	Intracapsular excision	Transcervical	+	−	1 + 1−	−

n.r., not reported.

Schwannoma of the cervical vagus is a rare entity generally causing only few, unspecific symptoms. Due to its benign nature and slow-growing rate, efforts to assure nerve preservation during surgical excision are mandatory, even if often very demanding. As recommended by most authors, careful intracapsular enucleation with nerve-sparing technique remains the treatment of choice in order to avoid nerve injury, even if complete nerve functionality cannot always be achieved [2,7].

In a review reported by Valentino *et al*., the authors state that complete excision can be performed, in cervical schwannomas, only in 56% of cases; of these, 64% showed permanent and 29% transitory neuronal deficit compared to 29% and 43%, respectively, in intracapsular enucleation or debulking technique [14]. Nevertheless, the very low recurrence rates reported in literature do not permit to formulate definitive conclusions on the preferable surgical technique [14-23].

The present review aimed to clarify if there is a difference in safety and efficacy among intracapsular approach and other surgical techniques in terms of complications and long-term outcomes on the treatment of cervical vagal schwannomas. Unfortunately, the rarity of this pathology does not allow a collection of data sufficient to reach our target. The consequence is a high heterogeneity of results, which, besides being too weak to elaborate a recommendation, could be even confusing in their interpretation.

## Conclusions

Schwannoma arising from the cervical vagus is a rare, benign pathology usually giving unspecific symptoms and requiring surgical excision, but frequently postoperative complications can affect patients lifelong, so, surgical indications should be based carefully on the balance between risks and benefits. Among surgical techniques, intracapsular enucleation seems to give better results in terms of preservation of nerve function, even if, in case of suspicion of malignancy, more extended resections should be needed, and vagal nerve resection and reconstruction should be considered.
